# Supplementary material for: A Glycosaminoglycan Extract from Portunus pelagicus Inhibits BACE1, the β Secretase Implicated in Alzheimer’s Disease
Source: Mar Drugs. 2019 May 16;17(5):293. doi: 10.3390/md17050293 (PMC6562973; doi:10.3390/md17050293)
Supplement: Supplementary file 1 [file marinedrugs-17-00293-s001.pdf]

**Supplementary Materials**

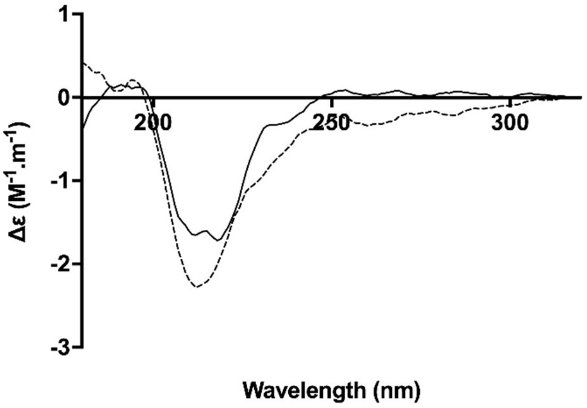


**S1:** The CD structural change of BACE1 (solid) observed in the presence of *P. pelagicus* F5 (dasded) with a ratio of 2:1 w/w, B:F5.
